# Functionalized 3D scaffolds for engineering the hematopoietic niche

**DOI:** 10.3389/fbioe.2022.968086

**Published:** 2022-08-17

**Authors:** Michela Bruschi, Tania Vanzolini, Neety Sahu, Alessandra Balduini, Mauro Magnani, Alessandra Fraternale

**Affiliations:** ^1^ Department of Biomolecular Sciences, University of Urbino Carlo Bo, Urbino, Italy; ^2^ Department of Orthopedic Surgery, School of Medicine, Stanford University, Stanford, CA, United States; ^3^ Department of Molecular Medicine, University of Pavia, Pavia, Italy; ^4^ Department of Biomedical Engineering, Tufts University, Medford, MA, United States

**Keywords:** hematopoietic niche, hematopoietic stem cells, megakaryocytes, platelets, 3D scaffold

## Abstract

Hematopoietic stem cells (HSCs) reside in a subzone of the bone marrow (BM) defined as the hematopoietic niche where, *via* the interplay of differentiation and self-renewal, they can give rise to immune and blood cells. Artificial hematopoietic niches were firstly developed in 2D *in vitro* cultures but the limited expansion potential and stemness maintenance induced the optimization of these systems to avoid the total loss of the natural tissue complexity. The next steps were adopted by engineering different materials such as hydrogels, fibrous structures with natural or synthetic polymers, ceramics, etc. to produce a 3D substrate better resembling that of BM. Cytokines, soluble factors, adhesion molecules, extracellular matrix (ECM) components, and the secretome of other niche-resident cells play a fundamental role in controlling and regulating HSC commitment. To provide biochemical cues, co-cultures, and feeder-layers, as well as natural or synthetic molecules were utilized. This review gathers key elements employed for the functionalization of a 3D scaffold that demonstrated to promote HSC growth and differentiation ranging from 1) biophysical cues, i.e., material, topography, stiffness, oxygen tension, and fluid shear stress to 2) biochemical hints favored by the presence of ECM elements, feeder cell layers, and redox scavengers. Particular focus is given to the 3D systems to recreate megakaryocyte products, to be applied for blood cell production, whereas HSC clinical application in such 3D constructs was limited so far to BM diseases testing.

## Introduction

Bone marrow (BM) is a complex microenvironment where several cellular elements co-habit and interconnect with each other and the extracellular matrix (ECM) for the maintenance of stem cells (Morrison and Scadden, 2014). Within the BM, the hematopoietic niche regulates the generation of blood and immune cells balancing between hematopoietic stem cell (HSC) quiescence, differentiation (hematopoiesis), and self-renewal. Oxygen tension (pO_2_) is not uniform throughout the BM, varying from oxygenated areas close to the sub-bone endosteal regions to more hypoxic zones towards the sinusoids, where HSC quiescence and survival are sustained (Wielockx et al., 2019; Congrains et al., 2021).

**FIGURE 1:**
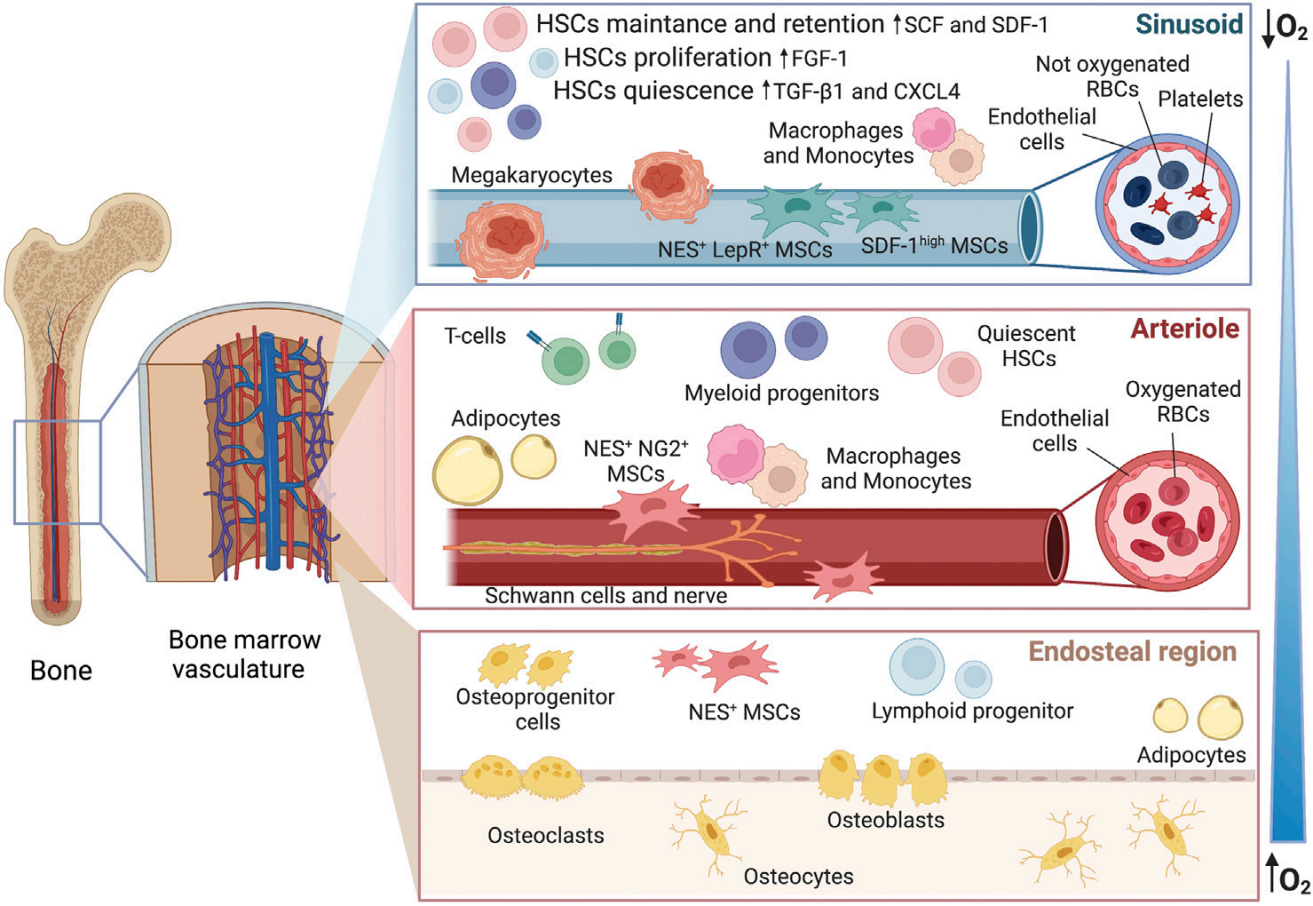
Hematopoietic niche within the bone marrow. The image offers an overview of the sub-regions with the gradient of oxygen, from hypoxic sinusoids to more oxygenated arterioles and endosteal area. *In vivo,* these micro-environments are all interconnected and the fate of HSCs is influenced by the secretome of the neighboring cells. As an example, megakaryocytes can release CXCR4 and TGF-β1 inducing quiescence, factors such as SDF-1 and SCF are released by the Nestin+ and SDFhigh perivascular cells leading to cell retention and self-renewal whereas FGF-1 from macrophages is conducting to proliferation. However, the action of multiple co-factors needs to be considered. CXCR4 platelet factor 4, FGF-1 fibroblast growth factor 1, SCF stem cell factor, SDF stromal cell-derived factor 1, TGF-β1 tumor growth factor-β1, NES nestin, NG2 nerve-glial antigen 2, LepR leptin receptor.

As illustrated in Figure 1, the niche hosts a variety of cells, which are not only HSC-derived cells (megakaryocytes, macrophages, regulatory T-cells) but also non-hematopoietic ones (as mesenchymal stromal cells, endothelial cells, osteoblasts, adipocytes, Schwann cells) (Pinho and Frenette, 2019; Dolgalev and Tikhonova, 2021), making the niche challenging to faithfully be reproduced it *in vitro*. Although HSCs constitute less than 0.01% of the total BM population, their multipotent role in hematopoiesis marks them as the cornerstone of the niche (Huang et al., 2007). Fine-tuning of cell-intrinsic factors such as transcriptional (e.g., GATA1 and PU.1) and chromatin regulators keep HSCs in a quiescent state in combination with extrinsic signals from the niche, e.g., hypoxia and growth factors (Rodrigues et al., 2021; Mann et al., 2022). Disruption of homeostasis due to danger-associated molecular patterns (DAMPs), proteolytic and lipolytic enzymes, inflammatory cytokines, and chemokines, i.e. IFNs, G-CSF, IL-1 ROS, suppresses quiescence and enforces HSCs proliferation (Pietras, 2017; Ratajczak et al., 2018).

Changes in regulatory mechanisms within the niche have a prominent role in the development of hematopoietic diseases. Aging of the BM micro-environment also favors disease progression. Age-related alterations involve the increased frequency of myeloid cells parallel to a decline of lymphopoiesis, red cell abnormalities like anemia, and a higher incidence of myeloid disorders, like myeloid dysplastic syndrome and acute myeloid leukemia (Pang et al., 2011; Skulimowska et al., 2021).

Currently, the only treatment for hematologic disorders is allogenic HSC transplantation. However, the limited number of available cells isolated from peripheral blood, umbilical cord, or BM is the major obstacle to their application (Zhou et al., 2020). Therefore, HSC proliferation, blood component production, and possible drug testing/toxicology are highly desirable in an *in vitro* system.

The challenge for the researchers is to create a bioengineered niche able to provide the specific cues needed for HSC expansion while preserving their stem cell properties and differentiation commitment into all the blood cell lineages (Rödling et al., 2017) considering the cellular heterogenicity and the sub-compartments found *in vivo* (Bessy et al., 2021).

**FIGURE 2:**
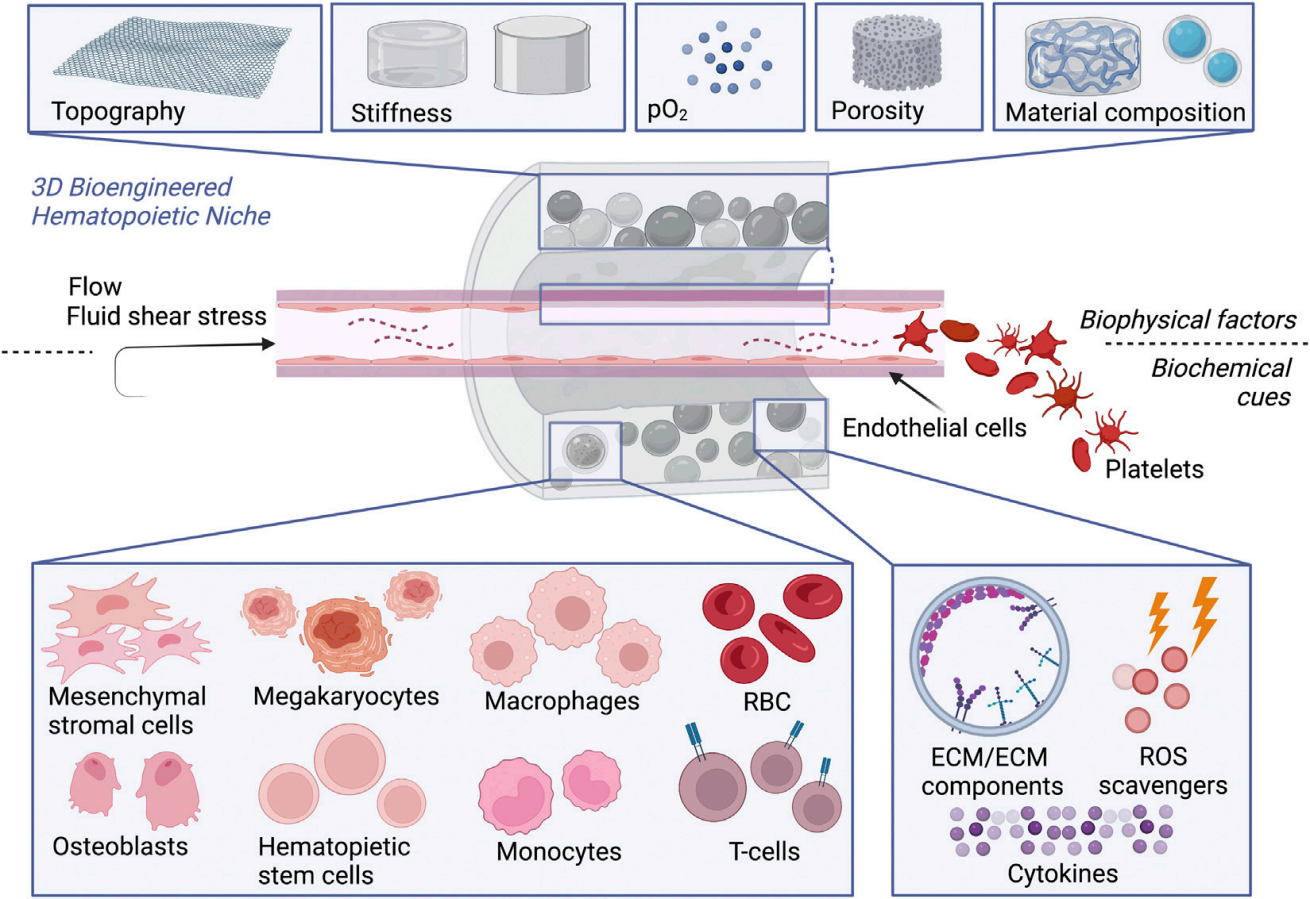
3D bioengineered hematopoietic niche *in vitro*. To promote HSCs expansion and differentiation or improve platelet production by megakaryocytes, scaffolds recreating the hematopoietic niche should offer simultaneously: 1) Physical cues, given by the composition of the biomaterial, topography, stiffness, oxygen tension, porosity, fluid shear stress. 2) Biochemical cues, provided either by the presence of cell monoculture or multi-culture or cellular components as ECM or ECM components (e.g., RGD peptides) to facilitate the homing of HSCs and in perfusion devices, by blood flow where RBC can be enrolled as carries for oxygen or bioactive molecules.

Hence, 3D cultures systems offer a better opportunity for such multi-cellular studies where a three-dimensional scaffold can provide biocompatibility (mimicking the extracellular matrix ECM), bioactivity (stimulating certain cytokines or specific gene expression), and biomechanical stiffness (comparable to the natural tissue for cell mechano-sensitivity) (Bruschi et al., 2015; Szcześ et al., 2017). Several materials have been tested; however, to achieve the required *stimuli*, functionalization of such scaffolds is necessary. In this review, we highlight the material properties, the modification needed to host HSCs, and further cell conditioning. A summary of all the factors involved in the modeling of a 3D niche is illustrated in Figure 2. Moreover, scaffolds for platelet generation from HSC-derived megakaryocytes will be described separately followed by the clinical outcomes of such 3D bioengineered constructs.

## Biophysical factors

The 3D architecture can be achieved *via* hydrogels where cells can be encapsulated, by fibrous scaffold, 3D printing, or by porous material (Lee-Thedieck and Spatz, 2012; Gaharwar et al., 2020). To mimic the natural niche, those scaffolds can be tuned with respect to: 1) topography; 2) stiffness 3) pO_2_ and 4) fluid shear stress (FSS).

The topography provides regulatory signals toward different cellular outcomes and the BM is influenced by the ECM (Jiang and Papoutsakis, 2013; Liu et al., 2022). The secreted ECM is mainly composed of structural proteins such as collagen I and IV, glycoproteins like fibronectin and laminin, and glycosaminoglycans (GAGs) (Matteini et al., 2021). The matrix organization varies within the niche showing a higher concentration of fibronectin and collagen type IV in the endosteal zone and a higher presence of laminin and collagen I in the perivascular region (Choi et al., 2015). ECM mimicry can be also achieved using electrospun nanofibers of nanometer size, porosity, and structure (Mahdavi and Enderami, 2022). Aside from the topography, HSC morphology is influenced by the matrix elasticity, consequentially their differentiation is strongly linked to the matrix stiffness (Zhang P. et al., 2019).

Stiffness is heterogeneous within the BM having ∼40 kPa in the endosteum closer to the bone cells, 10 kPa in marrow sinusoids, and 0.3 kPa in the central marrow (Janagama and Hui, 2020). The distribution of ECM components dictates the heterogeneity in BM stiffness. For instance, collagen type I increases the stiffness proportionally whereas collagen type III and type IV decrease it (Leiva et al., 2018).

*In vitro*, fibronectin-coated substrates (44 kPa) maintained the HSC myeloid progenitors and those laminin-coated (3.7 kPa) promoted differentiation towards erythroid lineages (Choi and Harley, 2017). Macrophage polarization is also affected by substrate stiffness, i.e. softer surfaces favored the shift to activated macrophages M1 whereas hard surfaces to macrophages M2 (Chen et al., 2020). Similarly, megakaryocytes cultured on soft gels (300 Pa) had higher ploidy, and greater pro-platelet formation than those cultured on stiffer gels (34 kPa) (Malara et al., 2011; Abbonante et al., 2017).

The interplay between stiffness and porous topography is linked.

Porosity (100–800 μm in diameter) and interconnectivity allow cell migration, distribution, and vascularization together with nutrient and oxygen supply within the material (Deschamps et al., 2017; Sari et al., 2021), a higher porosity increases the surface area favoring the absorption of growth factors. Zhou and co-workers designed a millimeter-scale porous scaffold for the encapsulation of feeder cells and a micron-scale for hosting HSC (Zhou et al., 2020).

Nevertheless, porosity affects the oxygen tension (pO_2_) within the scaffold, and HSC maintenance is increased in hypoxic conditions *in vitro*. In BM, the vascular zone has O_2_ tension of ∼12% but in the sinusoidal cavity O_2_ availability drops to ∼1–6% (Sayin et al., 2021).

Oxygenating biomaterials or carriers can be used to control oxygen levels and serve as 3D hypoxic environments (Fathollahipour et al., 2018). Park and Gerecht developed a hypoxia-inducible hydrogel absorbing oxygen *via* laccase, a multicopper oxidase that allowed pO_2_ levels and gradient to be tuned and predicted by reducing O_2_ to water molecules while concurrently oxidizing ferulic acid of the gelatin-ferulic acid-based hydrogel (Park and Gerecht, 2014). Furthermore, Zambuto and colleagues as well employed laccase to create a controllable hypoxic hydrogel (Zambuto et al., 2020). Other attempts to modulate the oxygen microenvironment involved polymeric carries with encapsulated hemoglobin or myoglobin as substitutes for RBCs (Willemen et al., 2021) However, materials with controlled hypoxia are still object of on-going investigations.

Blood perfusion can influence the activity of the cells in the niche despite not all of the cells are directly in contact with the blood flow (in sinusoids 0.2–0.8 mm/s) (Bixel et al., 2017). Fluid shear stress is absent in static culture systems despite this parameter can affect the hematopoietic niche through paracrine signaling stimulation, e.g. through Yap-1, Piezo 1 (Heck et al., 2020; Zhou et al., 2020; Li et al., 2021). Endothelial cells can convert FSS into a biochemical response for the neighboring cells, regulating cycling and quiescence of HSCs (Roux et al., 2020). FSS is necessary for megakaryocyte differentiation and maturation (Luff and Papoutsakis, 2016) as well as for the quiescence, maintenance, and apoptosis suppression of BM stromal cells (Zhang P. et al., 2019). In fact, the absence of blood circulation led to severe defects in hematopoiesis in both zebrafish and mouse embryos (Adamo et al., 2009; North et al., 2009).

## Biochemical *stimuli*

In the BM niche, HSCs interact with MSCs, which help maintain the HSC phenotype by the secretion of cytokines such as SCF, Flt3L, IL-6, and CSF-1 (Zhang et al., 2004). Nestin+ NG2+ MSCs are located in the periarterial niche, while Nestin+ LEPR+ and SDF-1high MSCs are observed in the perisinusoidal niche (Kunisaki et al., 2013; Kandarakov et al., 2022). Therefore, typically, 3D HSC culture would contain feeder cells such as mesenchymal stromal cells (MSCs) to facilitate the creation of a conducive microenvironment for the growth and sustenance of HSCs (Cook et al., 2012; Ferreira et al., 2018). While MSCs are predominantly used as feeder cells in the 3D culture of HSCs, other stromal cell lines such as umbilical cord endothelial cells and Wharton’s jelly cells are also used (Magin et al., 2009). Thus, 3D HSC cultures make use of primary stromal cells as a feeder for the long-term culture and expansion of HSCs. Braham *et al.* compared the efficacy of bioactive Matrigel and bioinert alginate hydrogels with the support of different feeder cells for the expansion of cord HSC progenitors (HSPCs) (Braham et al., 2019). The authors demonstrated that a combination of feeder cells consisting of MSCs differentiated toward adipocytes and osteoblasts along with endothelial progenitor cells (EPCs) self-assemble into a hypoxic stromal network, and therefore, better support HSPCs in the bioactive hydrogel.

As previously mentioned, in the HSC niche there are also ECM proteins, such as laminins, collagens, proteoglycans, and fibronectin which provide extrinsic cues that modulate HSC proliferation, differentiation, and migration, partly by providing anchorage for growth factors and cytokines such as tumor growth factor -β, interleukins, and other proteins. Thus, recreating the HSC niche *ex vivo* requires not only biophysical attributes but also biochemical functionalization to resemble the native archetype.

Different from utilizing feeder cells, 3D biomaterials functionalized by incorporation of ECM molecules or cell adhesion motifs are shown to recapitulate the HSC niche. Polycaprolactone-based 3D porous scaffolds coated with fibronectin (Mousavi et al., 2018) and vitronectin (Kim et al., 2019) (Kim et al., 2019) were shown to support and maintain the HSC phenotype. Incorporation of cell adhesion ligands derived from fibronectin instead of coating the entire fibronectin protein was shown to modulate HSC functions as well (Muth et al., 2013). Fibronectin has multiple binding sides or domains in its polypeptide chain for the adhesion of different growth factors (Rahman et al., 2005; Wijelath et al., 2006). Indeed, Cuchiara *et al* demonstrated that functionalization of PEG hydrogels with fibronectin-derived adhesive arginine-glycine-aspartic acid-serine (RGDS), showed superior adhesion of HSCs (Cuchiara et al., 2013). Retention signals involving the interaction between CXCR4 and VLA-4 receptors on the HSC surface with perivascular stromal cells expressing SDF-1 (also known as CXCL12), and vascular cell adhesion molecule 1 (VCAM-1), retain HSCs within the BM avoiding mobilization into the peripheral blood (Ratajczak et al., 2018). Méndez-Ferrer and colleagues demonstrated that the expression of SDF-1a is also regulated through circadian noradrenaline secreted by the sympathetic nervous system (SNS) which innervates the BM. The adrenergic signal is locally delivered by nerves and transmitted to Nestin+ MSCs by the b3-adrenergic receptor, leading to the rapid downregulation of SDF-1a and HSCs egress from BM (Méndez-Ferrer et al., 2008; Maestroni, 2020). Furthermore, the incorporation of stem-cell factor (SCF) and stromal-derived factor 1α (SDF1α) into the RGDS-PEG hydrogel further enhanced the adhesion and spreading of HSCs. SCF is known to regulate the HSC niche by promoting the survival and expansion of HSCs (Doran et al., 2009). Interestingly, Mahadik *et al.* also incorporated SCF in methacrylamide-functionalized gelatin (GelMA) hydrogels and demonstrated that SCF immobilization on the hydrogel improved selectivity of murine HSCs whereas soluble SCF promoted differentiation of HSCs in GelMA hydrogels (Mahadik et al., 2015).

Zhang *et al.* described an antioxidant hydrogel to circumvent the high levels of oxidative stress generated in *ex vivo* 3D cultures and mimic the low reactive oxygen species (ROS) levels in the HSC niche *in vivo* (Zhang Y. et al., 2019). They combined carbon nanotubes (CNTs), which can scavenge free radicals, with methacrylated hyaluronic acid to form hydrogels that can scavenge the culture system for oxides and peroxides generated during the culture of HSCs. In this system with low ROS levels, the proliferation ability and pluripotency of HSCs were improved as confirmed also by other groups (Cacialli et al., 2021). Thus, the biochemical composition of the HSC niche can be a blueprint for designing novel functionalization strategies of 3D hydrogels for recreating the HSC niche *in vitro*.

## Platelet generation

Investigations, about scaffold materials and the necessary *stimuli*, were performed for decades to develop efficient strategies to face the major problems related to BM engineered niches considering HSC scarce proliferation and the scale-up of blood components generation. Among the blood components, platelets are the most required because of their short lifespan which is further exacerbated by the inability to store them. Despite recent findings attributing an important contribution to platelets in inflammation and immune responses (Abbonante et al., 2020), the traditional and consolidated role of these anucleate cells concerns the maintenance of the hemostasis and thrombosis preventing bleeding by adhesion to injured vessels and thrombus formation (Thomas and Storey, 2015; Holinstat, 2017). In specific pathological or therapeutic conditions in which thrombocytopenia (low platelet count, < 150,000 per microliter of blood) occurs, the only available option to decrease mortality by bleeding is a transfusion. Unfortunately, the high demand of patients together with the platelet shortage, the donor-dependent issues, the cell quantity and functionality as well as the possible microbiological contamination, made urgent the need for platelet production (Abbonante et al., 2017; Vanzolini et al., 2022). HSCs represent suitable starting material since as a natural process, they differentiate directly in megakaryocytes which then mature in platelets. Alternatives consist of the direct differentiation of induced pluripotent stem cells (iPSCs) into megakaryocytes or their progenitors (Hansen et al., 2018; Shepherd et al., 2018; Nakamura et al., 2020) allowing the application of patient-derived cells and possible gene editing to address specific pathologies (Bruschi et al., 2021; Nakamura et al., 2021).

A 3D BM model made of silk fibroin derived from *Bombyx mori* silkworm cocoons was engineered to recreate the physiology of the human BM niche environment for the successful release of functional platelets into vascular tubes and study of megakaryopoiesis pathologies. Moreover, this silk platform represents a novel screening tool in the pharmaceutical industry to study the therapeutic effects of drugs on platelet number or function (Di Buduo et al., 2015). A significant improvement over the described silk model is a new miniature 3D model of human BM where megakaryocytes isolated from patient blood samples are linked to a scaffold made of silk and release platelets into an artificial bloodstream. This system proved useful to recapitulate *ex vivo* platelet biogenesis of patients and predict the platelet response to drugs, suggesting applicability for testing new compounds for inherited thrombocytopenia and other blood-related diseases, characterized by low platelet count and impaired hemostasis, for which effective treatment does not yet exist. (Di Buduo et al., 2021).

## Current clinical application

A model that successfully mimics the BM microenvironment under homeostasis and activated conditions would have various applications in the clinical and pharmaceutical fields. Research efforts have been addressed to engineer 3D BM models to study HSCs maintenance and hematopoiesis in steady-state and disease; for expansion of HSCs for transplantation, and as a platform for drug screening and toxicity testing in particular for myelotoxic effects of chemotherapeutics (Rödling et al., 2017). Several models have been developed during the last years for different clinical applications, from cancer pharmacotherapy to the production of large quantities of lab-grown blood cells for transfusion. Some examples are highlighted in this chapter.

Nelson and colleagues developed human BM-on-a-chip, incorporating endosteal, central marrow, and perivascular areas using osteogenic differentiated MSCs for mineralization, followed by MSCs and endothelial cells seeding over a fibrin-collagen hydrogel to model a 3D microvascular network favoring maintenance CD34^+^ HSCs (Nelson et al., 2021). Similarly, Glaser and co-workers utilized a microfluidic device to mimic the perivascular and endosteal niche allowing CD34^+^ maintenance and hematopoiesis as well. This model provided drug responses comparable to the *in vivo* ones (Glaser et al., 2022).

Furthermore, the BM niche sheds signals and provides cell-cell interactions supporting multiple myeloma. Myeloma cells alter the BM microenvironment to support tumor proliferation, resistance to therapy, cancer cell trafficking, and homing. Hence, different models are based on the culture of primary myeloma cells *in vitro*, in a 3D environment mimicking the human BM. The model, in which multipotent MSCs and their osteogenic derivatives were co-cultured with endothelial progenitor cells, facilitated the survival and proliferation of primary CD138+ myeloma cells for a few days (Braham et al., 2018). Furthermore, it was successfully used to test a novel class of engineered immune cells on primary myeloma cells providing a tool to investigate the interactions of primary myeloma cells within the BM niche and novel immunotherapies.

A 3D co-culture model composed of MSCs embedded in a hydrogel system and co-cultured with primary multiple myeloma patient cells has been used to study cellular components in the myeloma niche and the role of the BM microenvironment in the pathogenesis of multiple myeloma and drug resistance. In particular, this model was useful to reveal resistance to novel and conventional agents (Jakubikova et al., 2016).

Leukemic niche models have been engineered to investigate the role of the BM niche in the survival and uncontrolled proliferation of malignant leukemic cells (Raic et al., 2019). An example is given by the 3D PEG-heparin hydrogel with endothelial cells and MSCs developed by Bray and colleagues to investigate cell-cell interactions between vascular niche and leukemic cells and the response of leukemic cells to chemotherapeutics (Bray et al., 2017). These models could favor the transition of new drugs for hematological malignancies from pre-clinical to clinical research, as the lack of a suitable tumor microenvironment and/or BM architecture often impedes the clinical employment of promising drugs (Ramasamy et al., 2012). Moreover, clinical trials often fail due to a lack of drug efficacy, and no correlations between *in vitro* drug efficacy and clinical outcomes in hematologic malignancies have been described (Alhallak et al., 2021).

A 3D hydrogel system for hematopoietic differentiation of iPSCs has provided a tool for hematological disease modeling of Down syndrome-associated transient myeloproliferative disorder (TMD) which is a pre-leukemic stage present in 10–20% of children with trisomy 21 possessing the mutation in the transcription factor GATA-1 (Grimm et al., 2021). The model has permitted us to conclude that, in GATA1 mutant line, the erythroid population was reduced whereas the megakaryoid and myeloid populations were significantly increased, consistent with TMD characteristics (Sidhu et al., 2021).

In conclusion, 3D BM platforms provide tools for differential applications like studying patients’ cells to investigate disease mechanisms and identify personalized treatments as well as determining precisely the safety and efficacy of new drugs.

## Conclusion

Several efforts have been made to understand the intricate mechanisms within the hematopoietic niche and especially those concerning the fine-tuned homeostasis existing among HSC quiescence, self-renewal, and differentiation. Unfortunately, most of the attempts in culturing *ex vivo* or in reproducing *in vitro* the hematopoietic niche failed in being efficiently translated to the clinics. The main reason implicates the complexity of the microenvironments (endosteal, central medullary, arteriolar, and perivascular) in which HSCs harbor in the BM. These niches influence HSC behavior and fate thanks to different cues, interactions with other cell populations, and interestingly, tridimensional anatomical architecture. In particular, 3D structures seem to be essential in reproducing a system as close as possible to the original one avoiding polarization or phenotype as well as gene expression modifications, which is why they often replaced 2D cultures as study models (Jensen and Teng, 2020). Nevertheless, 2D systems are easier and more reproducible; moreover, based on the scaffold material, cell collection could be facilitated compared to tridimensional cultures. Hence, they are still preferred for large-scale production and high-throughput screenings (Bello et al., 2018; Varga et al., 2018). Moreover, some of the highly promising techniques are represented by microspheroids, organoids, and decellularized tissues (especially natural scaffolds) since they are the most realistic models and facilitate the engraftment of HSCs *in vivo* (Yin et al., 2016; Allenby et al., 2018; Harris et al., 2018; Janagama and Hui, 2020; Sahu et al., 2021).

Thus, understanding the behavior of HSCs in culture and their response to the cues of the artificial niche, could help with the success of their expansion *ex vivo* in 3D scaffolds (Tajer et al., 2019).

## Abbreviations

BM bone marrow; CXCR4 platelet factor 4; ECM extracellular matrix; FGF-1 fibroblast growth factor 1; Flt3 FMS-like tyrosine kinase 3; FSS fluid shear stress; HSCs hematopoietic stem cells; IL-6 interleukin 6; iPSCs induced pluripotent stem cells; MSCs mesenchymal stromal cells; PEG polyethylene glycol; RBC red blood cells; RGDS arginine-glycine-aspartic acid-serine; ROS reactive oxygen species; SCF stem-cell factor; SDF1α stromal-derived factor 1α; TGF-β1 tumor growth factor-β1; TMD Down syndrome-associated transient myeloproliferative disorder
